# Efficacy of sonic and ultrasonic irrigation devices in the removal of debris from canal irregularities in artificial root canals

**DOI:** 10.1590/1678-7757-2018-0045

**Published:** 2019-01-07

**Authors:** Gianluca Plotino, Nicola M Grande, Montse Mercade, Teresa Cortese, Simone Staffoli, Gianluca Gambarini, Luca Testarelli

**Affiliations:** 1 Sapienza Università di Roma, Department of Endodontics, Rome, Italy; 2 Università Cattolica del Sacro Cuore, Rome, Italy; 3 Universitat de Barcelona, Facultad de Odontología, Barcelona, Spain; IDIBELL Institute, Barcelona, Spain; IDIBELL Institute, Barcelona, Spain

**Keywords:** Disinfection, Root canal, Irrigation, Sodium hypochlorite, Ethylenediaminetetraacetic acid

## Abstract

**Objective:**

To evaluate the efficacy of different sonic and ultrasonic devices in the elimination of debris from canal irregularities in artificial root canals.

**Materials and Methods:**

A resin model of a transparent radicular canal filled with dentin debris was used. Five groups were tested, namely: Group 1 – ultrasonic insert 15.02; Group 2 – ultrasonic insert 25/25 IRRI K; Group 3 – ultrasonic insert 25/25 IRRI S; Group 4 – sonic insert 20/28 Eddy on a vibrating sonic air-scaler handpiece; Group 5 – 20.02 K-file inserted on a Safety M4 handpiece. Two different irrigants (5% sodium hypochlorite and 17% EDTA) and 3 different times of activation (20, 40, and 60 seconds) were tested. Means and standard deviations were calculated and statistically analyzed with the Kruskal-Wallis and Wilcoxon tests (p<0.05).

**Results:**

No statistically significant differences were found between the two irrigants used. Group 4 removed more debris than the other groups (p<0.05). Groups 1, 2, and 3 removed more debris than group 5 (p<0.05). A statistically significant difference (p<0.05) was found for the time of activation in all groups and at all canal levels, except between 40 and 60 seconds in group 4 at coronal and middle third level (p>0.05).

**Conclusions:**

No significant differences were found between 5% sodium hypochlorite and 17% EDTA. When the time of activation rises, the dentin debris removal increases in all groups. Both sonic and ultrasonic activation demonstrate high capacity for dentin debris removal.

## Introduction

Biomechanical preparation is known for being one of the key steps in root canal treatment.^1^ As the etiologic role of intracanal microorganisms is well-established in the development and advancement of periradicular and pulpal diseases, the fundamental goal of endodontic treatment is to eliminate all the pulp tissue and to disinfect the canal.^2^ ^,^ ^3^ Biofilms formed by bacteria are recognized to be present in unreachable areas of the root canal system,^4^ namely fins, accessory canals, and isthmuses. According to several studies, the mechanical instrumentation does not touch all the walls of the root canal^5^ ^,^ ^6^ and remaining biofilms and infected debris can be a possible source of persistent infection and treatment failure.^7^ For this reason, an adequate instrumentation and irrigation must be combined to decrease the microbial load within the root canal system and to complete the cleaning process.^8^

Different irrigating solutions have been used throughout the years and, among them, sodium hypochlorite has been the most used solution.^9^ Some concerns have been raised over sodium hypochlorite concentration and about the diffusion of the solution in some areas of the root canal, as complete root canal debridement has not been achieved.^10^ ^,^ ^11^ To improve the action of disinfection and debridement, different irrigation delivery devices are available, namely the use of sonic, ultrasonic and negative pressure devices.^12^ Agitation of sodium hypochlorite increases tissue dissolution^13^ and its continuous renewal affords an uninterrupted source of nascent chlorine for organic tissue dissolution.^14^

Most of the literature advises that ultrasonic devices are more powerful than sonic ones.^15^ Ultrasonic irrigation exhibits better canal debridement efficacy over the use of needle irrigation alone.^16^ Several *in vitro* and *in vivo* investigations studied the debridement efficacy of ultrasonic irrigation in the apical from 1 to 3 mm.^10^ ^,^ ^11^ ^,^ ^13^ ^,^ ^17^ ^,^ ^18^ However, ultrasonic irrigation presents some drawbacks; when the oscillating tip touches the root canal wall, for example, it dampens the energy and constrains the file movement, and file-to-wall contact occurs approximately 20% of the time.^19^ Moreover, ultrasonic files are made of metal alloy, therefore, when they touch the root canal wall, this may cause uncontrolled removal of dentin, deforming the root canal morphology.^20^

Among sonic devices, Endoactivator (Dentsply-Maillefer, Baillagues, Switzerland) is the most studied, but it operates only at approximately at 0.166–0.3 kHz and most of the studies showed better results for ultrasonic irrigation, probably because of the higher power (approximately 40 kHz).^21^ ^–^ ^24^

Recently, a new sonic system has been introduced into the market, the Eddy system (VDW GmbH, Munich, Germany), which is driven at a frequency of 6000 Hz by an air-driven handpiece (SONICflex 2003 Airscaler, Kavo, Genova, Italy). The manufacturer claims that the high-frequency vibration produced is transferred to the polyamide tip, which is moved in an oscillating movement at high amplitude thanks to the original qualities of the material. This three-dimensional movement generates “cavitation” and “acoustic streaming” – two physical effects which have only been known to be triggered by passive ultrasonic irrigation (PUI).^25^

Currently, there are no publications regarding the effectiveness of the Eddy system in penetrating canal irregularities. The aim of this study is to evaluate the efficacy of different sonic and ultrasonic devices in the elimination of debris from canal irregularities in artificial root canals.

## Material and methods

### Model used to reproduce dentinal debris

**Figure 1:**
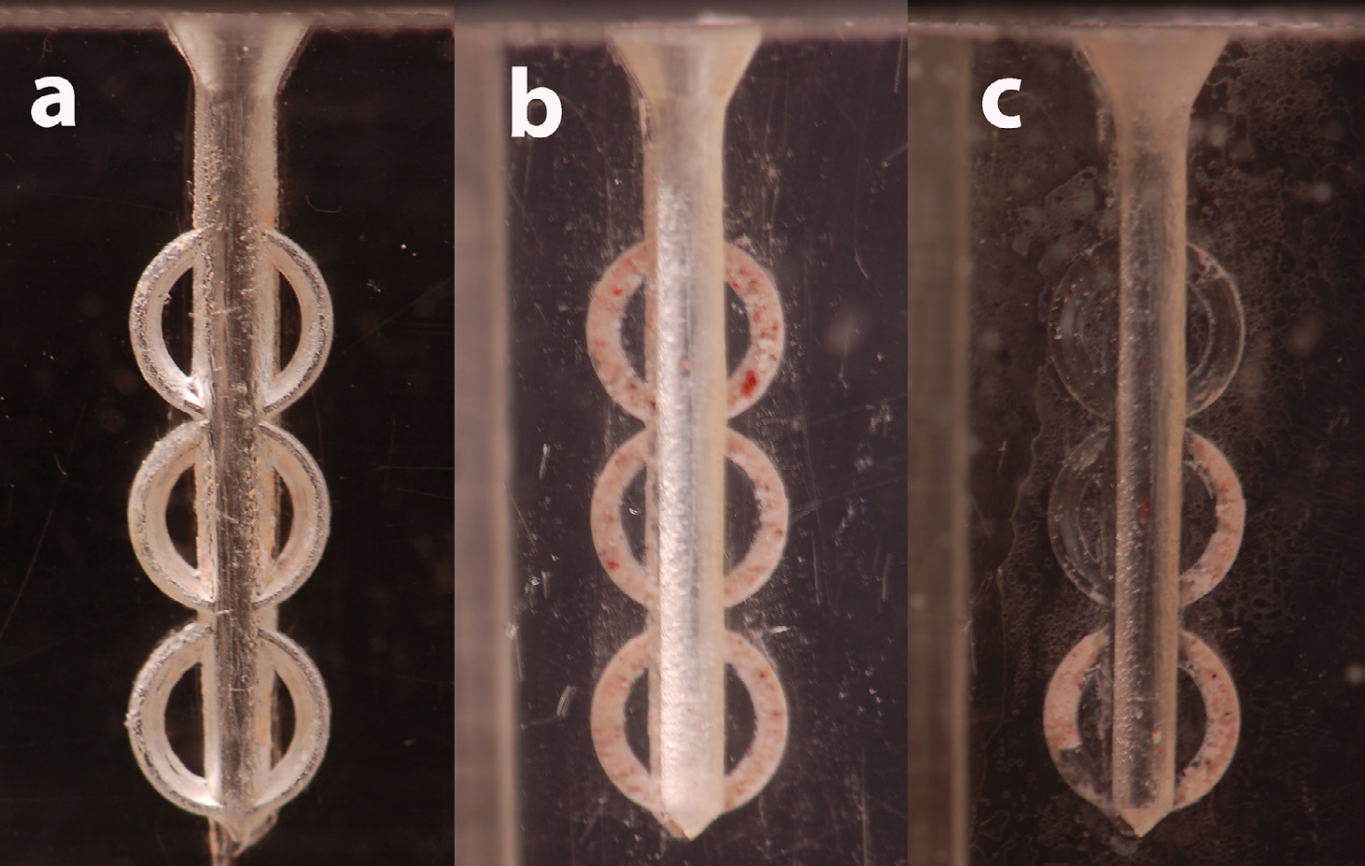
The study model used in this study (a); the same model with all the lateral extensions filled with dentin debris (b); an example of a sample with residual debris in few areas after the treatment (c)

The study was conducted on a transparent resin model of the radicular canal, divided into two parts of equal thickness (1.2 mm each) (Figure 1). The dimensions of the radicular canal resin model were 10 mm length and 2.5 mm width. The surface of both sections showed a depression, with the same measures, placed in the same position, so that once assembled (by means of two screws) each depression overlapped to its counterpart to reproduce the lumen of a root canal. To simulate the presence of lateral canal irregular extensions, 3 semi-circular cavities were done in the surface of one of the two sections for each side of the simulated canal, in the coronal, middle, and apical section, respectively.

The three semicircles *per* side were filled with dentin debris which simulated debris accumulated in non-instrumented areas of the root canal, obtained from extracted teeth using coarse-grained sandpaper, weighted with an electronic scale and mixed with water to obtain a compound similar to wet sand, drying it for 5 seconds with an absorbent paper.

### Experimental groups

The same simulator of the root canal was employed for all the groups tested and the test was repeated 10 times for each experimental group. Each time, the same irrigation procedure was adopted, using inserts of various shapes and sizes, activated in a different manner or with different frequencies of oscillation. The same operator performed all experimental procedures.

Five experimental groups were defined:

Group 1: ultrasonic insert 15.02, 40 kHz of oscillation frequency (EndoUltra - Vista, Racine, USA);
Group 2: ultrasonic insert 25/25 IRRI K, 28-36 kHz of oscillation frequency (VDW GmbH, Munich, Germany);
Group 3: ultrasonic insert 25/25 IRRI S, 28-36 kHz of oscillation frequency (VDW GmbH, Munich, Germany);
Group 4: sonic insert 20/28 Eddy, 6 kHz of oscillation frequency (VDW GmbH, Munich, Germany) on a vibrating sonic air-scaler handpiece (ZA-55 - W&H Bürmoos, Austria);
Group 5: 20.02 K-file inserted on a Safety M4 handpiece, <6 kHz of oscillation frequency (Sybronendo, West Collins Orange, USA).

### Irrigation procedure

The empty simulated root canal was completely filled for all experimental groups with 5% NaOCl (Ogna, Muggiò (MB), Italy), by means of a 3 ml Luer-lock sterile syringe, with a 27-gauge endodontic needle (Navi Tip, Ultradent, Utha, USA) placed 1 mm from the working length (WL). The activated file/tip was inserted 1 mm shorter to the WL and centered in the canal to reduce contact with the walls. Subsequently, it was activated for 20 seconds and the procedure was repeated for two further 20-second cycles, each time using new sodium hypochlorite to fill entirely the main simulated root canal; therefore, the total irrigant activation was 1 minute. This procedure was repeated 10 times for each experimental group.

The experiment was then repeated, using 17% EDTA as irrigant instead of 5% sodium hypochlorite to also evaluate the influence of the liquid used in the removal of debris within the lateral extensions of the simulated canals.

### Image evaluation and statistical analysis

The root canal model was photographed with a digital camera (Nikon D50, Tokyo, Japan) before the test (with the lateral extensions filled with dentin debris) and after each irrigation cycle of 20 seconds, totaling 4 pictures *per* sample. The resulting images were viewed and automatically analyzed using the AUTOCAD software (AutoCAD® 2012, Autodesk, San Rafael, USA).

The first image was taken to confirm that all lateral extensions were filled with dentine debris, and to calculate the total area of the lateral extensions filled with dentine debris and consisting in the sum of the six semicircles present. In addition, the initial debris-filled area was calculated considering couples of semicircles in relation to their position in the coronal, middle, and apical part of the simulated canal (A1=coronal, A2=medium, A3=apical). The area occupied by the debris was marked in each image made after each irrigant activation cycle (20/40/60 seconds) (Figure 1). The areas filled by debris were calculated before and after each of the 3 cycles of irrigation and the percentage of debris removal at each stage was obtained as follows:

$$\text{percentage of removed debris}=\frac{\text{area before irrigation}-\text{area after irrigation}}{\text{area before irrigation}}\times 100$$

Percentage of debris removal was also calculated as a function of the position of the lateral extensions in the simulated root canal (coronal, middle, apical).

The differences in the ratios of removed debris between groups were analyzed by the Kruskal-Wallis and Wilcoxon tests. The level of significance was set at p=0.05.

## Results

Table 1 shows the percentage of debris removal for each experimental group.

**Table 1:** Percentage of debris removal for each experimental group ± standard deviation (SD). Considering the total section, same superscript letters (“a,” “b,” or “c”) on the same line indicate no statistically significant differences. Considering the total section, same superscript letters (“x,” “y,” “w,” or “z”) on the same column indicate no statistically significant differences. Same superscript letter “d” means no statistical difference among thirds in the same time of activation in the same group. Same superscript letter “e” means no statistical difference among time of activation in the same group. The groups that do not have any letter do not have significant differences with the other groups

	G1: EndoUltra		G2: Irri K		G3: Irri S		G4 Eddie		G5 M4	
Irrigants	NaOCl	EDTA	NaOCl	EDTA	NaOCl	EDTA	NaOCl	EDTA	NaOCl	EDTA
**Coronal third**										
20 sec	40.06±14.88^a^	43.61±13.88	47.24±14.90	47.02±13.58	59.73±18.51	54.33±15.40	93.51±14.59	92.71±10.72	29.42±14.71	30.22±12.16
40 sec	64.70±14.13	65.42±13.87	74.76±17.62	75.32±13.06	82.06±13.83	80.34±12.22	99.07±2.93	99.24±2.03	50.00±15.18	48.08±13.45
60 sec	77.05±13.80	76.90±10.32	86.56±9.94	85.05±10.07	93.38±9.93	83.07±10.33	99.32±4.48	99.30±2.04	66.04±11.39	66.78±10.98
**Medium third**										
20 sec	34.43±15.39	36.12±13.77	41.68±9.56	44.03±10.12	47.38±15.85	44.90±13.55	84.73±13.51	85.33±10.19	24.60±11.42	26.12±12.30
40 sec	57.20±8.25	56.45±10.42	65.44±14.03	65.98±10.76	62.15±13.23	60.11±10.88	97.87±4.09	97.90±3.83	44.06±10.53	45.56±8.12
60 sec	68.77±4.83	70.44±8.37	78.73±6.69	80.48±8.09	68.67±11.89	73.09±11.89	99.03±3.06	98.88±2.56	57.85±16.84	60.13±13.64
**Apical third**										
20 sec	31.14±16.96	34.12±18.47	32.48±8.16	35.88±10.22	34.27±16.89	32.22±16.81	47.84±17.93	50.04±18.88	22.51±12.52	22.35±11.49
40 sec	52.97±16.90	53.87±14.71	53.51±14.92	56.32±12.87	42.04±15.72	46.64±10.02	81.26±19.69	80.16±18.46	42.48±12.66	42.08±12.22
60 sec	70.34±16.47	69.61±13.96	70.96±11.23	73.66±13.33	63.07±5.84	68.86±8.22	97.86±4.63	97.08±2.05	60.08±15.47	57.05±13.14
**Total**										
20 sec	35.18±13.69^ax^	38.56±9.28^ax^	40.36±8.44^ax^	42.09±11.68^ax^	46.98±8.44^ax^	41.63±11.54^ax^	74.93±9.94^ax^	75.09±9.34^ax^	25.49±12.24^ax^	28.13±14.71^ax^
40 sec	58.25±9.33^ay^	61.08±12.56^ay^	64.44±12.79^ay^	65.07±10.53^ay^	61.86±11.69^ay^	63.16±12.97^ay^	92.54±7.54^ay^	92.95±4.33^ay^	45.50±11.82^ay^	44.48±9.02^ay^
60 sec	72.08±9.31^az^	73.32±11.21^az^	78.66±6.86^az^	80.38±14.63^az^	75.00±7.57^az^	76.60±7.17^az^	98.73±2.83^az^	98.55±2.69^az^	61.36±12.40^az^	63.46±11.12^az^

No statistically significant differences have been found between the two different irrigants used, at all levels and intervals of activation (p>0.05).

Concerning the total amount of debris removed, group 4 has statistically removed more debris than the other groups (p<0.05). Moreover, group 1, 2, and 3 statistically removed more debris than group 5 (p<0.05), whereas there were no differences among group 1, 2, and 3 (p>0.05). No statistical difference was found among group 4 after 20 seconds of activation and groups 1, 2 and 3 after 60 seconds of activation (p>0.05).

The more the time of activation increased, the more debris was eliminated. A statistically significant difference (p<0.05) was found for the time of activation (20 x 40 x 60 seconds) in all groups and at all canal levels, except between 40 seconds and 60 seconds in group 4 at coronal and middle third level (p>0.05).

Lateral extensions of the artificial canal at coronal level resulted in an statistically better removal of debris than middle and apical thirds (p<0.05), except for group 4 (p>0.05). No differences were found between the middle and apical levels in all groups (p>0.05).

## Discussion

The aim of this study was to evaluate the efficacy of different sonic and ultrasonic devices in the elimination of debris from canal irregularities in artificial root canals filled with sodium hypochlorite or EDTA. The results showed no statistically significant differences between NaOCl and EDTA, therefore, it seems that the mechanical movement of the liquid is more important than the chemical action for removal of debris.

The predominant irrigation method among endodontists seems to be passive ultrasonic irrigation (PUI).^26^ To the date, most studies showed favorable results for PUI compared to sonic irrigation.^15^ PUI has some advantages, namely the acoustic streaming effect that increases wall shear stress and enhances the rupturing of intra-radicular biofilm.^27^ However, PUI has also some drawbacks. First of all, the contact of the file with the root canal walls dampens the energy of the oscillating instrument and constrains the file movement.^9^ This is an important limitation in curved root canals because the file stops and cannot oscillate freely. Moreover, ultrasonic files, although having a non-cutting tip, are made of steel, and steel is harder than dentin, so ultrasonic tips could deform the root canal and are only recommended as a final irrigation.^28^

The results of this study revealed no significant differences among the different ultrasonic inserts used, despite their differences in dimensions and type of the tips.

Marketed sonic devices until present showed lower results than ultrasonic devices, mainly due to their lower power. Typically, a sonic device operates at 1-8 kHz and ultrasonic at 25-40 kHz.^9^ ^,^ ^29^ Sonic devices present some advantages regrding ultrasonic ones: the oscillating points are made of a plastic-like material, it does not stop when in contact with the root canal wall, and it is not able to deform the root canal, so it can be used safely in curved root canals.

Eddy system has been recently launched to the market, claiming for a much more power than other sonic devices. Results of this study indicated that Eddy performed better than all the other groups at all time intervals and at all root canal levels, corroborating the results of a recent article that concluded that passive sonic irrigation with Eddy system at 6000 Hz might be at any rate similar to PUI regarding the decrease of bacteria in curved and straight root canals^25^, as well as those from two other recent articles that concluded that activation with EndoActivator, Passive Ultrasonic Irrigation, and Eddy increased the tissue dissolving activity of irrigants from artificial grooves in root canals of extracted teeth.^30^ ^,^ ^31^

In this study, 3 activation times of 20 seconds each were used, according to the clinical protocol suggested previously.^16^ Significant differences were found in each group at different time intervals: as a general rule, the greater the time of activation, the greater the cleanliness. However, if clinicians take a look at the Eddy group, the 20-second activation with Eddy was similar to the 60-second ultrasonic agitation. Moreover, there were no significant differences between 40 seconds of sonic irrigation with Eddy and 60 seconds, so a reduction in clinical time may be advised.

The *in vitro* model used in this study has some limitations, being an artificial root canal with artificial extensions simulating inaccessible areas of the main root canal. Dentin debris was obtained from fresh teeth and packed into the lateral extensions as reported previously.^32^ The walls in these plastic systems are smooth and regular, thus different to the dentin surface. The lateral extensions are quite large, cylindrical, and placed along a cylindrical simulated root canal; maybe for that reason, many statistical differences among thirds were not found, because there is no difference in dimensions among the levels of activation. Furthermore, the usage of pictures only analyzes a two-dimensional area of the canal. However, this *in vitro* method is useful for standardizing the amount of debris accumulated and the amount of irrigant introduced in all groups tested. Further clinical and laboratory studies are needed to evaluate the Eddy system efficacy.

## Conclusions

No statistically significant differences were found between 5% sodium hypochlorite and 17% EDTA activation and among the ultrasonic inserts used. When the time of activation rises, the dentin debris removal increases in all groups. Both sonic and ultrasonic activation demonstrate a good capacity for dentin debris removal. The Eddy sonic system removed more debris from lateral extensions than the other systems tested.
